# Nutrient-dependent interactions between a marine copiotroph *Alteromonas* and a diatom *Thalassiosira pseudonana*

**DOI:** 10.1128/mbio.00940-23

**Published:** 2023-09-29

**Authors:** Guanjing Cai, Xiaoqi Yu, Hui Wang, Tianling Zheng, Farooq Azam

**Affiliations:** 1 Biology Department and Institute of Marine Sciences, College of Science, and Guangdong Provincial Key Laboratory of Marine Biotechnology, Shantou University, Shantou, China; 2 State Key Laboratory of Marine Environmental Science and Key Laboratory of the Ministry of Education for Coastal and Wetland Ecosystems, School of Life Sciences, Xiamen University, Xiamen, China; 3 Marine Biology Research Division, Scripps Institution of Oceanography, University of California, San Diego, La Jolla, California, USA; University of Tennessee at Knoxville, Knoxville, Tennessee, USA

**Keywords:** bacteria-diatom interaction, nutrient dependence, chemotaxis, competition, algicidal bacteria

## Abstract

Bacteria-diatom interactions in the ocean are diverse but usually studied in static conditions, which limits our understanding of their importance in marine ecosystems and biogeochemical cycles. Here, we explored the dynamic interactions between an ubiquitous marine bacterium *Alteromonas* sp. and a diatom *Thalassiosira pseudonana* under different nutrient conditions. In oligotrophic conditions, minor shifts in nutrients qualitatively altered the interactions from mutualism during early exponential growth to weak parasitism during the late stationary phase. Organic matter-activated *Alteromonas* chemotactically swam toward, attached on, and dramatically broke down *T. pseudonana* cells, leading to an aggressive parasitic behavior with a 95% algicidal rate. Meanwhile, inorganic matter-activated *T. pseudonana* showed amensalism against *Alteromonas*, resulting in an ephemeral decrease of bacterial abundance by 27%. Interestingly, when both organics and inorganics were sufficient, *Alteromonas* suppressed diatom growth by inhibiting the cell division, while the surviving *T. pseudonana* restored proliferation with a significantly smaller cell size inconducive to bacterial attachment, demonstrating an intense competition. The results further indicated that the algicidal effect of *Alteromonas* was controlled by the cell-specific protease activity and the number of attached bacteria on the diatom cell surface, both of which were related to nutrient conditions. Since the nature and intensity of bacteria-diatom interaction depend on the composition and richness of nutrients, it mechanistically explains the tripartite relationship among bacterial proliferation, nutrient viability, and algal demise during blooms. The algicidal behavior of copiotrophs also potentially enhances the contribution of a microbial carbon pump to carbon sequestration in the ocean.

**IMPORTANCE:**

As the major producers and consumers, phytoplankton and bacteria play central roles in marine ecosystems and their interactions show great ecological significance. Whether mutualistic or antagonistic, the interaction between certain phytoplankton and bacterial species is usually seen as a derivative of intrinsic physiological properties and rarely changes. This study demonstrated that the interactions between the ubiquitously co-occurring bacteria and diatom, *Alteromonas* and *Thalassiosira pseudonana*, varied with nutrient conditions. They overcame hardship together in oligotrophic seawater but showed antagonistic effects against each other under nutrient amendment. The contact-dependent algicidal behavior of *Alteromonas* based on protease activity solved the paradox among bacterial proliferation, nutrient viability, and algal demise haunting other known non-contact-dependent algicidal processes and might actually trigger the collapse of algal blooms *in situ*. The chemotactic and swarming movement of *Alteromonas* might also contribute greatly to the breakdown of “marine snow,” which could redirect the carbon sequestration pathway in the ocean.

## INTRODUCTION

Phytoplankton, as the main primary producers in the ocean, supply organic matter to the marine food web and therefore are responsible for a substantial fraction of the downward flux of organic matter in the ocean (1). Meanwhile, bacteria in the phycosphere are among the major consumers of phytoplankton-derived organic matter, which also channel the biomass from other marine creatures into the microbial loop and regenerated inorganic nutrients to in turn support the growth of phytoplankton (2). Considering their ubiquity and dominance in the ocean, the material exchange and energy flow between bacteria and phytoplankton form the major framework of the marine ecosystem and profoundly influence all marine creatures in the ocean (3), which highlights the ecological necessity of studying phytoplankton-bacteria interactions in the phycosphere.

Phytoplankton-bacteria interactions vary from mutualism to parasitism, which are controlled by their intrinsic physiological properties, as well as environmental conditions (4): mutualism is usually based on nutrient exchange (5) and occurred in oligotrophic conditions (6); competition for nutrients is also commonly reported in nutrient-limited environments (7); antagonism is recognized with the discoveries of numerous algicidal bacteria (8) and phytoplankton-derived antibiotics (9) under nutrient-rich cultivable conditions. Since the physiological status of phytoplankton and bacteria can be variable, the interactions between specific species are also not static. A “Jekyll-and-Hyde” interaction, which describes the shift from mutualism to antagonism, has been discovered between roseobacters and phytoplankton. The studies showed that the changes of interactions greatly depended on the growth phases of either phytoplankton (aging) (10) or bacteria (quorum sensing) (11). Besides the intrinsic variation of physiological status during growth, the environmental condition, e.g., the availability of substrates (12), is also decisive for the growth of phytoplankton and bacteria and therefore might fundamentally change their interactions. However, the correlation between phytoplankton-bacteria interaction and environmental condition is rarely discussed.

In this study, targeting on the ubiquitous co-occurrence of globally distributed marine plankton *Alteromonas* and *Thalassiosira* demonstrated by a previous study (13) and our *in situ* samples, we analyzed the influences of nutrient conditions on their interactions at different temporal and spatial scales. Their fine-tuned behaviors could provide clues for the coevolutionary interactions of bacteria and diatoms in changing marine environments.

## MATERIALS AND METHODS

### Sources of bacterial and diatom strains

A fast-growing bacterial strain (named L15) was isolated from a seawater sample taken from the coastal algal bloom area off Xiamen, China (118°10′30″E, 24°34′27″N), in 2015. 16S rRNA gene sequencing showed that L15 was most closely related to *Alteromonas macleodii* ATCC 27126^T^ (Fig. S1). It was preserved on Zobell agar plates (peptone 5 g/L, yeast extract 1 g/L, ferric phosphate 0.1 g/L, agar 12 g/L, dissolved in seawater filtered by GF/F filters, Whatman, and pH 7.6–7.8) and to get the seed culture. Axenic *Thalassiosira pseudonana* CCMP 1335 (abbreviated as *Tp* hereafter) was purchased from the National Center for Marine Algae and Microbiota (NCMA). To get the seed cultures for further experiments, L15 was cultivated in Zobell liquid medium (without agar) for 18 h (180 rpm, 20°C), while *Tp* was cultured in sterilized F/2 medium under a light intensity of 40 µmol photons m^−2^ s^−1^ with 12 h:12 h light-dark cycle at 15°C (same light and temperature conditions were also applied in the following experiments) for 7 d.

### Experimental setups for long-term co-cultivation

L15 and *Tp* cells in the seed cultures were collected by centrifugation at 3,000 × *g* for 5 min, washed twice, and resuspended in filtered autoclaved seawater (FASW, taken from the Scripps Pier, 117°15′26″W, 32°52′6″N). After SYBR Green I (1×) staining for 5 min, cell densities were measured by a flow cytometer (BD Accuri C6 plus) to calculate the volume of inocula for co-cultivation.

Co-cultivation experiments were carried out using FASW and F/2 as basal media, respectively (Fig. S2). In FASW-based culture, L15 and *Tp* were inoculated together each with an initial cell density of 10^4^ mL^−1^ (marked as “Co-culture”) and the monocultures were treated as the control groups (marked as “L15” and “Tp”, respectively). At 48 h, aliquots of these three experimental groups were amended with 0.1% Zobell medium (vol/vol, marked as “L15 + Z/1,000,” “Tp + Z/1,000,” and “Co + Z/1,000,” respectively). In F/2-based culture, L15 and *Tp* were inoculated together at initial cell densities of 5 × 10^6^ and 10^4^ mL^−1^, respectively, and supplemented with (marked as “Co* + Z/100”) or without (marked as “Co-culture*”) 1% Zobell medium (vol/vol). Correspondingly, monocultures of *Tp* supplemented with and without 1% Zobell medium were marked as “Tp* + Z/100” and “Tp*,” respectively. Meanwhile, three potential mechanisms by which bacteria inhibited diatom growth were tested (14, 15): (i) to test whether bacteria secreted metabolites with algicidal activity, 1% filtrate of L15 culture was added into the monoculture of *Tp* and marked as “Tp* + FS/100”; (ii) to test whether bacteria with quantitative superiority suppressed diatom growth due to nutritional competitive advantage, co-cultures with different initial bacteria/diatom ratios were set up (details concerning cell densities were shown in the legend of Fig. S9); and (iii) to test whether bacteria directly attached to diatom cells and manipulated diatom growth, samples were additionally analyzed for attached bacteria numbers and diatom cell lengths, as well as total cell counts (supplementary materials). All groups were triplicated. Variation of nutrient concentration (NO_3_^−^, NO_2_^−^, NH_4_^+^, PO_4_^3−,^ and DOC) in the oligotrophic seawater was also measured (supplementary materials).

### Observation of the motility and chemotaxis of L15 cells

Following a published method (16, 17), a polydimethylsiloxane (PDMS)-based microfluidic device was fabricated and casted on the silicon mold provided by Jeffrey S. Guasto lab, Tufts University, which is schematically shown in Fig. S3A. The device was then bonded to a glass slide and filled with FASW through inlet A and B at 100 nL/min. For experiments, the fluid from inlet A was replaced with a suspension of L15 (grown for 18 h, washed, and resuspended in FASW at 10^9^ mL^−1^, left still for 3 h) while either FASW (negative control), TP7, TP15 (cell-free filtrates of *Tp* grown for 7 and 15 d, respectively), or 1% Zobell medium (vol/vol, positive control, named Z/100) was injected through inlet B. The fluids were then gradually slowed down until they were completely still. The movement of bacteria was then analyzed for swimming velocity and chemotactic enrichment (supplementary materials).

In addition, a suspension of L15 (same as above) amended with 0.1% (vol/vol) Zobell medium was injected through inlet A and a suspension of *Tp* (grown for 15 d, 10^4^ mL^−1^ in FASW) was inject through inlet B. Fluids were slowed down gradually, and a single *Tp* cell laid at the center of diatom fluid far away from the bacterial fluid was targeted as the center of field of view (Fig. S4). The whole procedures were replicated four times. The chemotactic movement of bacteria toward each diatom cell was then measured (supplementary materials).

### Observation of the attachment of L15 on *Tp* cells

A suspension of *Tp* (10^6^ mL^−1^ in FASW) was added into Nunc Lab-Tek Chamber Cover Glass and amended with 0.1% (vol/vol) Zobell medium. After settlement, *Tp* cells were observed under a Nikon A1R confocal microscope (bright field with 20× objective). Ten microliters suspension of L15 was then carefully dropped next to the field of view, avoiding the displacement of settled *Tp* cells. One minute later, pictures were taken at 0.1 fps for 17 min and merged into a time-lapse video. Real-time videos were also recorded at 1 and 10 min.

To test the integrity of *Tp* cells, a suspension of *Tp* was first stained with fluorescein diacetate (FDA) at 8 µg/mL for 5 min without additional wash before being added into the chamber. Since the sterility of the chamber could not be guaranteed in an open environment, L15 cells were distinguished by staining the suspension with DAPI at 1 µM for 10 min and washed with FASW once before being inoculated as the above. Pictures were taken at 0.033 fps for 60 min under confocal mode with 60× objective and merged into a time-lapse video. To rule out cell rupture or fluorescence quenching caused by laser irradiation, FDA-stained *Tp* cells without L15 addition were observed under the same condition as control. Videos were generated and processed with Adobe Premiere Pro 2022.

### Measurement of the protease activity of L15 cells

Aliquots of “L15” and “Co-culture” as described above were amended with 1% Zobell medium (vol/vol, marked as L15 + Z/100 and Co + Z/100, respectively). An aliquot of “Co + Z/100” was also added with 1 mM phenylmethylsulfonyl fluoride (PMSF, a serine-protease inhibitor (18), marked as “Co + Z/100 + PMSF”). Samples were collected at 3 h and 48 h and filtered through 5- and 0.22-µm filters sequentially in order to separate the free-living and attached bacteria. Aliquots of the filtrates were stained with DAPI and counted for the abundances of L15 and *Tp* under the epifluorescence microscope, while other aliquots of the filtrates were measured for the protease activity using L-Leucine-7-amido-4-methylcoumarin as the substrate (final concentration of 50 µM) following the protocol described before (19). Cell-specific protease activity was then calculated (supplementary materials).

### Measurement of the physiological properties of *Tp* cells

*Tp* cells were first stained with 1 µM carboxyfluorescein diacetate succinimidyl ester (CFDA-SE) for 30 min at 20°C and then washed with FASW twice. L15 and pre-stained *Tp* were inoculated together into Zobell-amended F/2 medium (1%, vol/vol) at initial cell densities of 5 × 10^6^ and 10^5^ mL^−1^, respectively. The same medium only inoculated with pre-stained *Tp* was treated as a control group. Samples were taken at 3, 48, 72, and 144 h and measured by flow cytometry. The APC signal was used to screen *Tp* cells, which were recorded for forward scatter channel (FSC) and FITC signals simultaneously. *Tp* cells were also collected by filtration and measured for cell lengths.

Meanwhile, unstained *Tp* cells were co-cultured with L15 as the above conditions, sampled at 3, 48, 72, and 144 h, and incubated with PDMPO (LysoSensor Yellow/Blue DND-160, (2-(4-pyridyl)−5-((4-(2-dimethylaminoethylaminocarbamoyl)methoxy)phenyl)oxazole) for 24 h at a final concentration of 0.125 µM under the same cultivation condition. PDMPO binds to polymerizing Si and emits an intense fluorescence under ultraviolet (UV) excitation (20). The FITC signal of APC-screened *Tp* cells was recorded by flow cytometry. Data were processed by FlowJo X 10.0.7.

All data were preliminary processed by Excel 2021, statistically analyzed by SigmaPlot 14.0, and plotted by Origin 2022.

## RESULTS

### Shift of interactions between L15 and *Tp* in the oligotrophic seawater

**Fig 1:**
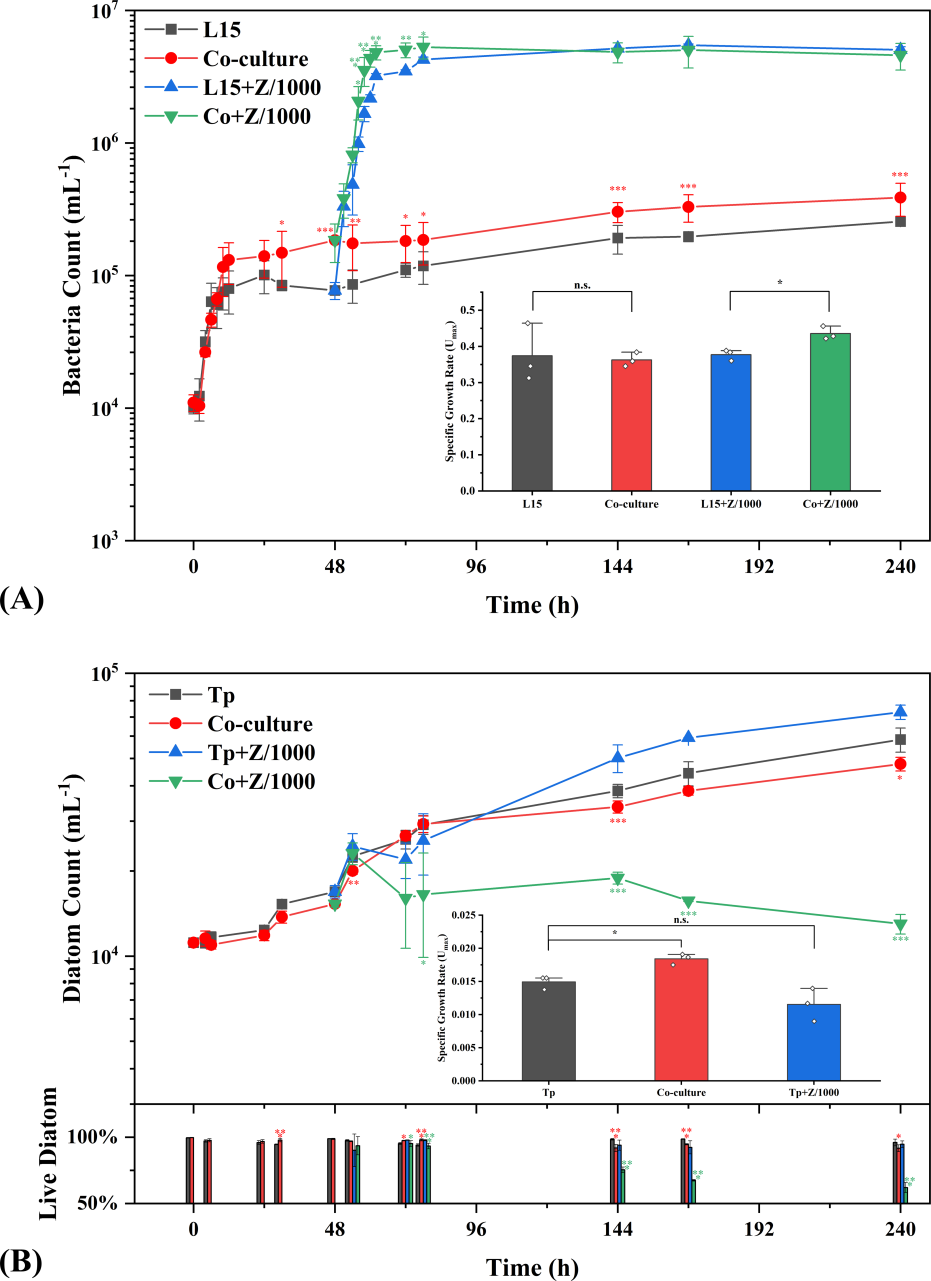
Growth of L15 (**A**) and *Tp* (**B**) in FASW-based cultures. Inserted charts show the specific growth rates calculated based on the Gompertz model. The down chart shows the percentage of live diatom. L15/Tp, monoculture of L15/*Tp* in FASW; Co-culture, co-culture of *Tp* and L15 in FASW; Co + Z/1000, co-culture of *Tp* and L15 in FASW amended with 0.1% Zobell medium. The red or green asterisk represents that Co-culture or Co + Z/1000 is statistically different from monoculture of L15/Tp or L15/Tp + Z/1000 (*t*-test, *0.01 < *P* < 0.05, **0.001 < *P* < 0.01, and ***P < 0.001).

In a previous study, we analyzed the bacterial community compositions of 25 *Thalassiosira* samples *in situ* and confirmed the highest frequency of occurrence of *Alteromonas* in the phycosphere, especially *T. pseudonana* samples, all of which contained relatively high abundances of *Alteromonas* (~1%, Fig. S5). Similar with the *in situ* habitats, we first tested the growth of L15 and *Tp* in the oligotrophic seawater. The results showed a gradual shift from mutualism to weak parasitism. In the coculture, the cell count of L15 was constantly higher than control after 30 h (Fig. 1A, *P* < 0.05, *t*-test) and *Tp* experienced a significantly faster exponential growth characterized by the 1.23 times higher specific growth rate (*P* < 0.05, inserted chart in Fig. 1B; Fig. S6); afterwards, the population of L15 was maintained higher, while the abundance of *Tp* lagged behind control (Fig. 1B, *P* < 0.05 at 144 and 240 h) and showed a higher mortality (down chart in Fig. 1B, *P* < 0.05). The variation of their growth matched the changes of nutrients in the seawater (Fig. S7): the high-abundance L15 in the coculture consumed more dissolved organic carbon (DOC, *P* < 0.05); the assimilatory of nitrogen (the consumption of nitrate and ammonium minus the production of nitrite) and phosphorus (the consumption of phosphate) by *Tp* in the coculture was higher than control before 72 h but slowed down during 72−144 h. Nevertheless, the interactions between L15 and *Tp* seemed to be mild in the oligotrophic seawater since neither burst growth nor collapsing population was observed.

### Intensified interactions between L15 and *Tp* with amendment of organics or inorganics

**Fig 2:**
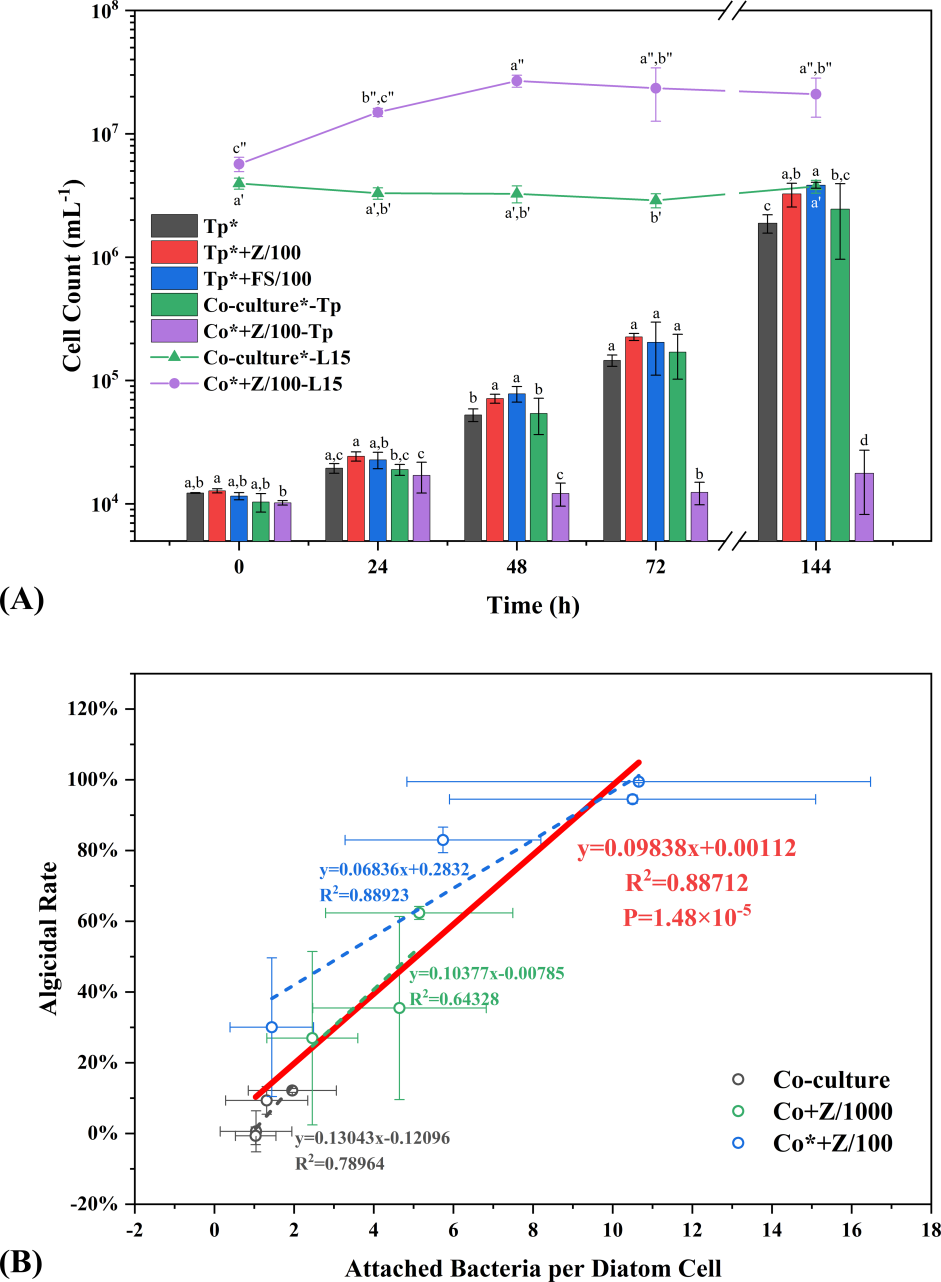
Growth of L15 and *Tp* in F/2-based cultures (**A**) and linear fits between attached bacteria per diatom cell and algicidal rates (**B**) based on the samples from “Co-culture” (black), “Co + Z/1000” (green), “Co* + Z/100” (blue), and all of them (red). Tp*, monoculture of *Tp* in F/2 medium; Tp* + Z/100, monoculture of *Tp* in F/2 medium amended with 1% Zobell medium; Tp*+FS/100, monoculture of *Tp* in F/2 medium amended with 1% filtered supernatant of L15 culture; Co-culture*, co-culture of *Tp* and L15 in F/2 medium; Co* + Z/100, co-culture of *Tp* and L15 in F/2 medium amended with 1% Zobell medium. Columns at the same time points marked with different letters are statistically different from each other. Letters with single apostrophe show the statistical difference among different time points in Co-culture*-L15. Letters with double apostrophe show the statistical difference among different time points in Co* + Z/100-L15 (*P* < 0.05, one-way ANOVA, Duncan’s multiple range test).

The physiological activities of L15 and *Tp* were then enhanced by selective amendment of nutrients. L15 boosted by 0.1% Zobell medium (mainly organic nutrients) showed a strong antagonistic effect on *Tp*, which experienced a collapse of population and a spike in mortality (the green line and column in Fig. 1B), resulting in a 95% algicidal rate at 240 h. Meanwhile, L15 benefited from the attack on *Tp*, judging from the significantly higher specific growth rate (*P* < 0.05, inserted chart in Fig. 1A), which demonstrated a typical parasitism. Similarly, *Tp* grown in F/2 medium (mainly inorganic nutrients) decreased the abundance of L15 by 27% during the first 72 h (Fig. 2A, compared with 0 h, *P* < 0.05, Duncan’s multiple range test). However, *Tp* did not significantly benefit from the demise of L15 and L15 restored initial abundance at 144 h, resulting in an ephemeral amensalism. Nevertheless, the results showed that the balance between L15 and *Tp* would be greatly tilted by biased nutrient amendment. The beneficiary from nutrient amendment would be also the dominator in bacteria-diatom relationship.

Further, when organics and inorganics were amended simultaneously (F/2 medium with 1% Zobell), the growth of *Tp* was severely suppressed by the proliferating L15, resulting in a constantly low abundance around 10^4^ mL^−1^. In turn, the surviving *Tp* also suppressed the growth of L15, judging from the prolonged exponential growth (48 h in the co-culture vs ~24 h in the monoculture shown in Fig. S8). In addition, the resistance of *Tp* usually emerged as the co-cultivation progressed, especially when the initial cell density of *Tp* increased. The low bacteria/diatom ratio (1 × L15 + 3 × Tp in Fig. S9) helped *Tp* restore rapid proliferation at 144 h (5.74-fold of the abundance at 48 h, *P* < 0.05, Duncan’s multiple range test), causing a simultaneous demise of L15 at 144 h (88% of the abundance at 48 h, *P* < 0.05). It indicated that an intense arms race, namely, competition, would occur between L15 and *Tp* when they were both activated by appropriate nutrients.

Summarizing the results of long-term co-cultivation experiments, it seems that the algicidal effect of L15 against *Tp* was attachment related. The three independent groups with different nutrient amendments at different time points showed similar trends: the average number of attached bacteria per diatom cell (Fig. S10) had a strong positive correlation with the corresponding algicidal rate (Fig. 2B). It also suggested that organic amendment increased the algicidal rate by enhancing the capability of L15 to attach to *Tp* cells.

### Induced chemotactic movement of L15 led to leaky *Tp* cells

**Fig 3:**
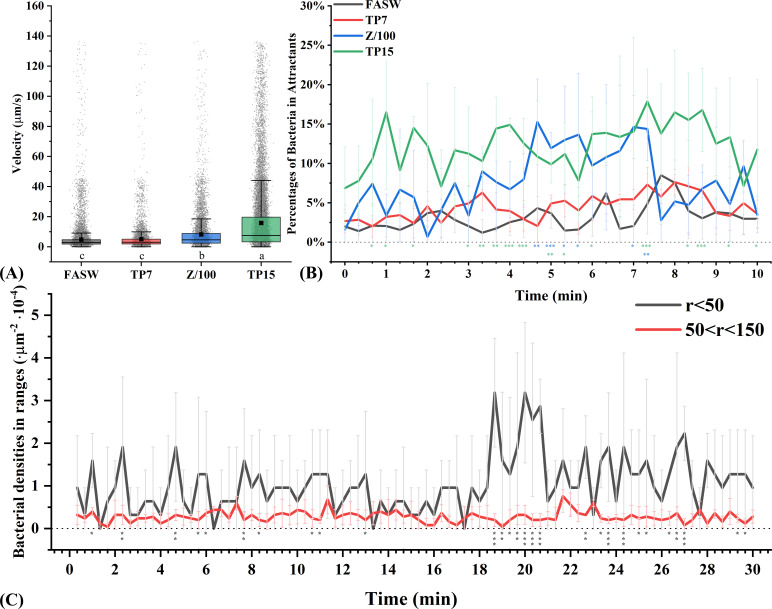
Swimming velocities of L15 (**A**) in FASW, 1% Zobell medium (Z/100), cell-free filtrates of *Tp* culture grown for 7 (TP7) and 15 d (TP15), and the percentages of L15 attracted by different fluids (**B**) and *Tp* cells (**C**). Groups marked with different letters in A are statistically different from each other (*P* < 0.05, one-way ANOVA, Duncan’s multiple range test). Asterisks with the corresponding colors in B represent statistical differences from FASW. Asterisks in C represent statistical differences between *r* < 50 and 50 < *r* < 150 (*t*-test, *0.01 < *P* < 0.05, **0.001 < *P* < 0.01, and ***P < 0.001).

The attachment on *Tp* cells was then found to be based on the chemotactic movement of L15. First, the swimming velocity of L15 could be boosted by aged *Tp* culture (Fig. 3A). As negative and positive controls, the average velocity of L15 was only 4.76 µm/s in FASW (Fig. S11A) and increased to 8.20 µm/s in Z/100 (Fig. S11B, *P* < 0.05, Duncan’s multiple range test). In comparison, the cell-free filtrate of 7-day-old *Tp* culture (TP7) had minor effect on the average velocity of L15 (5.15 µm/s, Fig. S11C), while L15 cells in the 15-day-old *Tp* filtrate (TP15, 15.87 µm/s, Fig. S11D) swam even faster than those in Z/100 (*P* < 0.05). It was reasonable since diatoms under stress, e.g., aged *Tp* cells in this study, released more DOM (21) that could greatly manipulate bacterial metabolism (22). Second, L15 swam toward aged *Tp* culture. Using FASW as a baseline, less than 10% of L15 cells would swim in the fluid of attractant due to random movement (the black column/line in Fig. 3B; Fig. S11E). The percentages of L15 in TP7 were as low as those in FASW, while Z/100 attracted significantly more L15 cells during the 4 min 40 s to 7 min 20 s interval (*P* < 0.05, the blue column/line in Fig. 3B; Fig. S11E). In addition, TP15 attracted L15 even more strongly: statistically higher percentages of L15 in TP15 (*P* < 0.05) were recorded at more than half of the time points (16 of 31). Finally, L15 could sense a single *Tp* cell. As shown in Fig. 3C; Fig. S11F, L15 cells far away from the *Tp* cell (the outer circle 50 ~ 150 µm from the center) showed a stable and low density. Compared with them, L15 cells close to the *Tp* cell (the inner circle < 50 µm from the center) were just occasionally more abundant during the first 18 min (10 of the 54 time points with *P* < 0.05), then accumulated dramatically during the 18 min 40 s to 20 min 40 s interval (*P* < 0.05), and maintained at a higher level after 21 min (11 of the 27 time points with *P* < 0.05). The results clearly demonstrated that L15 would chemotactically swim in response to the gradient of the exudates/lysates of *Tp*.

**Fig 4:**
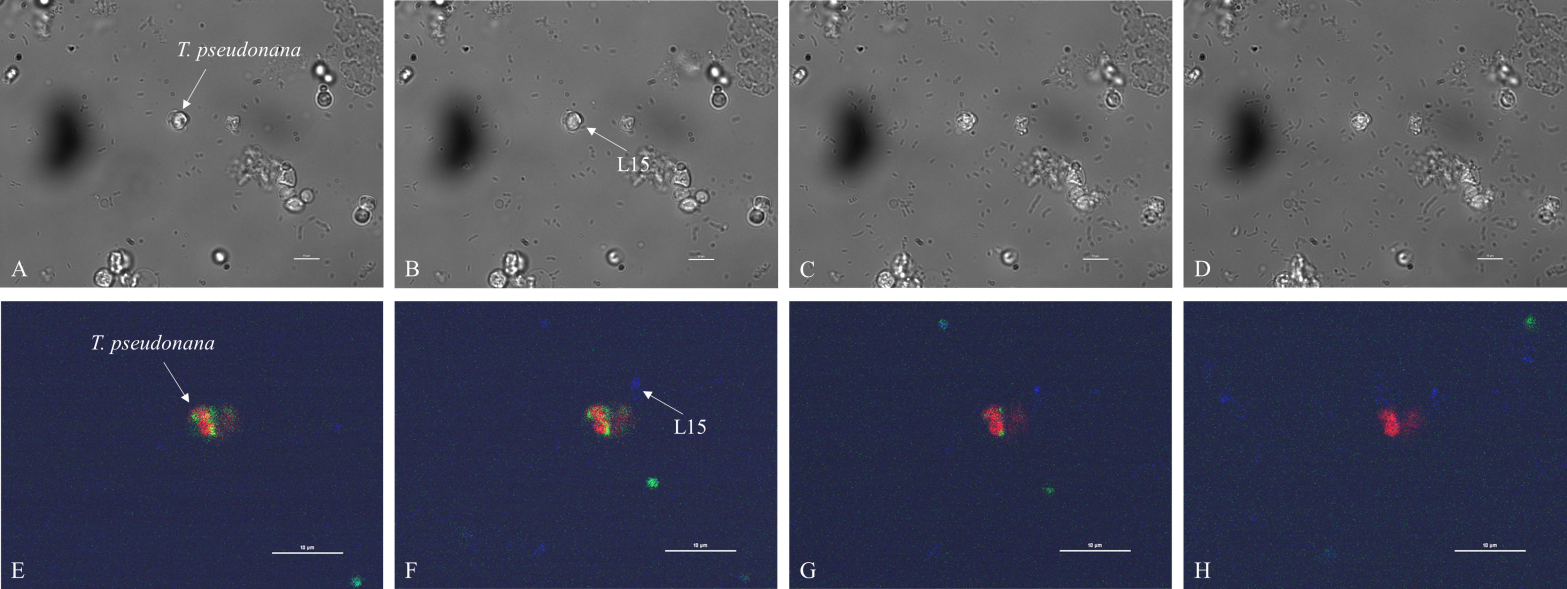
Short-term effects of the attachment of L15 on the morphology (A, 1 min; B, 4 min; C, 8 min; and D, 15 min) and integrity (E, 1 min; F, 20 min; G, 40 min; H, 60 min) of *Tp* cells. Blue, DAPI; green, fluorescein; red, chlorophyll.

Microscopic videos then revealed the fast impact of L15 on *Tp* cells. First, the morphology of the *Tp* cell changed significantly within minutes, as shown in a time-lapse video (Video S1) focusing on a *Tp* cell (marked with an arrow). The cell changed from a three-dimensional shape (Fig. 4A and B) to a “flat” state within only 8 min (Fig. 4C) and then shrank continuously, leading to a final accumulation of more bacteria around it (Fig. 4D). The bacterial accumulation could be also observed in a real-time video (Video S2): only a few bacteria wriggled around *Tp* cells 1 min after the addition of L15; much more bacteria swam quickly and colonized on *Tp* cells just 10 min later. Then, FDA staining demonstrated that the *Tp* cell would become leaky after the attachment of L15. Fluorescein, produced when FDA was hydrolyzed intracellularly, should be well contained by intact *Tp* cells (Tp + Z/100 in Video S3). However, once the surface of a *Tp* cell was attached by a L15 cell at 20 min (marked with an arrow in Fig. 4F), its signal of fluorescein began to fade out 10 min later (Fig. 4G) and disappeared at 40 min (Fig. 4H), indicating the breakdown of cell integrity. The 20 min interval between bacterial attachment and diatom rupture was similar to what was observed in Fig. 3B (~19 min). The morphological changes of *Tp* cells could even occur in less than 10 min, suggesting the aggressiveness of L15 attack.

### A protease-dependent algicidal behavior of L15

**Fig 5:**
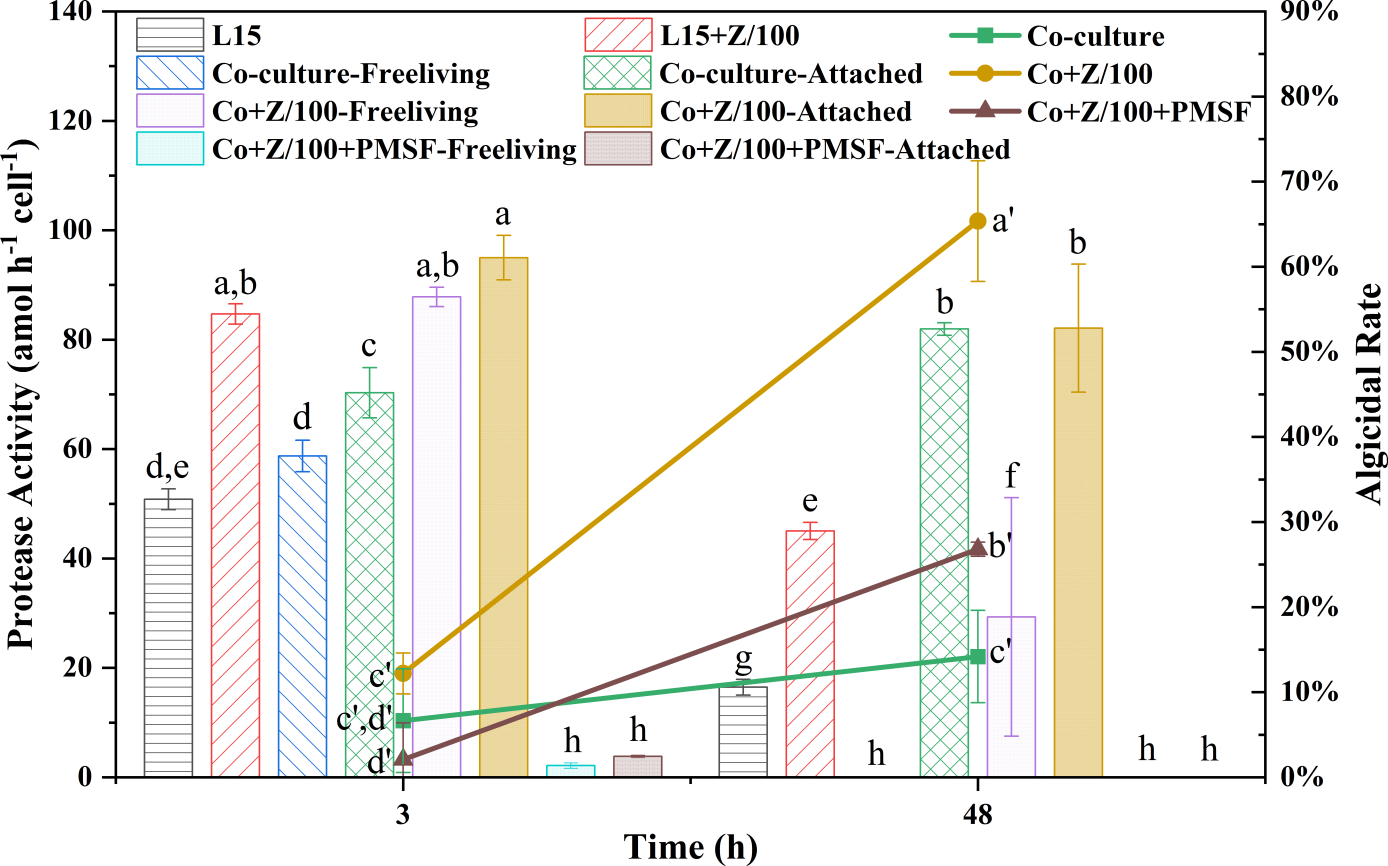
Protease activities of free-living and attached L15, as well as algicidal rates under different treatments. Columns of protease activities marked with different letters are significantly different from each other. Letters with a single apostrophe show statistical differences among algicidal rates (*P* < 0.05, one-way ANOVA, Duncan’s multiple range test).

As for how L15 made *Tp* cells leaky, the frustule protein could be a potential breakthrough point. Besides crystalline quartz and pectin, protein is also a major component of diatom frustule (23), which made protease an effective algicidal compound (15, 24). In this study, adding protease inhibitor PMSF reduced the algicidal rate from 65.4% to 26.8% at 48 h (*P* < 0.05, Duncan’s multiple range test, Fig. 5), indicating the dependence of L15 on extracellular proteases to break down *Tp* cells. Analysis on the reference genomes of *A. macleodii* and *Tp* further showed that >80% of the extracellular proteases (33, 35, and 38 of 40, Table S1) of *A. macleodii* had potentials to cleave the top 80% amino acids that made up the frustulins of *Tp* (FRU1, FRU2, and FRU3, Table S2), which were glycoproteins that constituted a protective coat covering all parts of frustule (25). Among the proteases, WP_014951274 and WP_014949348 are even known for hydrolyzing cell wall glycoproteins, which makes the fast breakdown of *Tp* cells reasonable. In addition, the protease activity of L15 was highly correlated with the availability of organic sources. Free-living L15 could not maintain high protease activity at 48 h, while attached L15 were constantly active, whether in FASW or Zobell-amended culture. It suggested that chemicals on the cell surface of *Tp* could be important cues for the protease activity of L15 and explained the high correlation between the algicidal rate and number of attached bacteria in the oligotrophic seawater.

### Variation of *Tp* cell division and size during L15 attack

**Fig 6:**
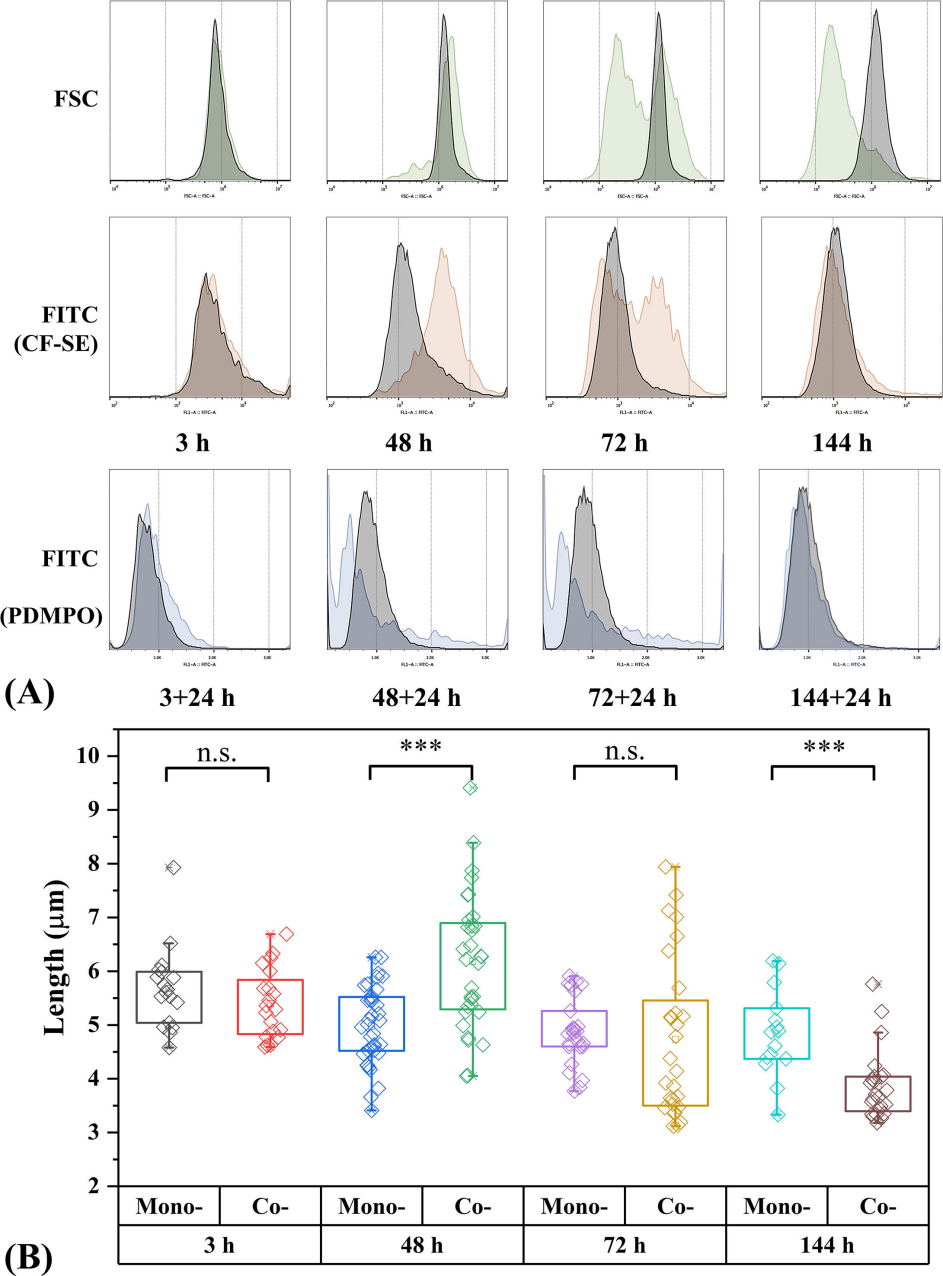
Flow cytometry analysis (**A**) of the cell size (signal of forward scatter channel [FSC]), cell division (FITC signal of remained CF-SE), and silica deposition (FITC signal of PDMPO, measured after 24 h of incubation) of *Tp* grown in monoculture (the gray peaks) and in co-culture (the green, orange, and blue peaks) and microscopic measurement of the lengths of diatom cells (**B**). Significance detection between monoculture and co-culture is performed using *t*-test (ns: *P* > 0.05, ***P < 0.01).

Besides disrupting cell integrity, the attack of L15 also interfered *Tp* cell division. *Tp* cells in the monoculture divided normally, leading to decreasing CF-SE signals since 48 h (Fig. 6A). In comparison, *Tp* cells in the co-culture divided differently: most cells contained high CF-SE at 48 h, suggesting an arrest of cell division; then, the signals of some cells dropped at 72 h, resulting in two distinct populations; finally, the peak overlayed with that of the monoculture at 144 h, indicating the full restoration of cell division. Variation of cell division in the co-culture could be verified by PDMPO staining that showed weaker silica deposition/frustule synthesis at 48 + 24 h and 72 + 24 h, which restored at 144 + 24 h. The arrest of cell division further caused the enlargement of *Tp* cells. Besides the slightly higher frustule synthesis at 3 + 24 h and FSC signal at 48 h, microscopic measurement indicated that the average cell length of *Tp* increased from 5.0 to 6.2 µm (*P* < 0.001, Fig. 6B), which benefited the growth of bacteria (26). Interestingly, after the restoration of cell division, *Tp* cells in the co-culture became much smaller, judging from the dramatic change of FSC peak and the 1-µm shorter average cell length. It could be an effective survival mechanism of *Tp* adapting to the attack of L15, since size reduction of *Tp* would increase the chance of escaping the attachment of L15 (Fig. S12).

## DISCUSSION

Although both our data and previous studies (13) showed that the co-occurrence of *Alteromonas* and *Thalassiosira* was ubiquitous, their relationship, as a good representative for studying bacteria-diatom interactions, was rarely discussed. Our study showed that *Alteromonas-Thalassiosira* interactions tended to shift from mutualism to weak parasitism under the oligotrophic condition. Nutrient variation in the seawater revealed some interesting phenomena. As shown in the monoculture, the slow-growing *Tp* in the oligotrophic seawater did not release more DOC (Fig. S7). It suggested that the fast-proliferating L15 in the coculture was more likely to benefit from the bound extracellular organic carbon of *Tp*, which was sufficient in supporting the mutualistic bacteria in the phycosphere (27). It also indirectly highlighted the importance of cell attachment in mediating *Alteromonas-Thalassiosira* interactions. Meanwhile, the variation of *Tp* growth could be highly correlated with the constant ammonium depletion in the co-culture caused by the competition of L15. On one hand, it relieved the suppression on nitrate assimilation (28), allowing *Tp* to utilize a more plentiful nitrogen source (10–12 µmol/L of nitrate vs 0.1–0.25 µmol/L of ammonium) from the very beginning for a faster growth. On the other hand, less limitation on nitrate utilization meant less stress on growth. It might lead to lower sexual production of *Tp* cells (29), and long-term asexual production could cause clonal death that capped final abundance (30). It implied that the shift of interactions in the oligotrophic seawater was more likely to be regulated by an energy-saving strategy based on fine manipulation of nutrients.

Mild interactions under the oligotrophic condition would turn into escalated antagonistic effects against each other under eutrophic conditions. Similar effects of an *Alteromonas* strain on a marine cyanobacterium were reported before: it enhanced the growth of a specific strain of *Prochlorococcus* (10^6^ mL^−1^) at low cell densities (10^5^ and 10^6^ mL^−1^) and yet inhibited the cyanobacterium at a higher concentration (10^7^ mL^−1^) (31). The “Jekyll-and-Hyde” interaction could be also observed in some other bacteria-phytoplankton co-culture systems (10, 11). However, all these studies described the antagonistic effect just from a bacterial perspective and did not mention the capability of phytoplankton to inhibit bacteria under different conditions. They also emphasized the regulatory effects of infochemicals but ignored environmental factors that might fundamentally alter the behaviors of bacteria and phytoplankton *in situ* (32). Focusing more on ecological significance, our study additionally described the amensalism of *Thalassiosira* against *Alteromonas* and indicated that the composition and richness of nutrients determined the nature and intensity of the bacteria-diatom interaction. They shared “hardships” but not “joys,” which was less like the known “Jekyll-and-Hyde” interaction but more similar to the “hunger game” hypothesis describing interbacterial relationships under different nutrient supplies (33).

The antagonistic effect of *Alteromonas* against *Thalassiosira* was determined as contact dependent. Despite that some *Alteromonas* were known for secreting algicidal compounds (34 – 36), the filtrate of L15 culture had no negative effect on the growth of *Tp* (Tp*+ FS/100 in Fig. 2A). The hypothesis that bacteria have nutritional competitive advantage over diatom through quantitative superiority was also ruled out since the growth of *Tp* was almost unaffected by the high initial bacteria/diatom ratio without Zobell amendment [4 × L15 + 1 × Tp, similar yield of Zobell-activated L15 after 3 h (1 × L15 + 1 × Tp + Z/100), Fig. S9]. Microscopic observation indicated that the algicidal behavior of L15 was initiated by the highly responsive movement toward diatom exudates/lysates. It could be related to the selective utilization of DOM components (37) or diatom-derived signal molecules that regulated bacterial behavior (38). The chemotactic movement of L15 toward a diatom cell also significantly escalated after 18 min 40 s (Fig. 3C), which was similar with the bacterial response to lysing diatom cells (39). Combined with the characterization by FDA staining, the results revealed fast physiological changes of *Tp* cells under the attack of L15. Further analysis revealed that the protease activity of L15 contributed to the attack greatly. However, it should be noted that complete suppression of protease activity by PMSF could not guarantee complete elimination of algicidal effect. The incomplete restoration of *Tp* growth might suggest the involvement of additional activities in the algicidal process (24), or be caused by the biogenic silica decreasing due to PMSF addition (40). Anyway, the protease activity established the baseline of algicidal behavior, since each attached bacterial cell showed a similar activity level regardless of organic amendment; the chemotactic movement decided the multiplier for algicidal effect, considering its positive correlation with the number of attached bacteria. Therefore, exogenous organic matter could regulate algicidal intensity by controlling the number of attached bacteria that decided the bulk protease activity. The nutrient-dependent algicidal behavior could helped *Alteromonas* better adapt to a changing environment, leading to the dominance in both oligotrophic ocean (41) and bloom areas (42).

Besides causing cell leakage, the protease-dependent algicidal behavior also explained how *Alteromonas* manipulates the cell size of *Tp*. Genomic analysis suggested that the extracellular proteases of *A. macleodii* could effectively degrade silacidin (Table S2), the deficiency of which caused a significant increase in the valve diameter of *Tp* (43). Meanwhile, it could explain the smaller newly divided *Tp* cells after they survived the bacterial attack: the decrease of silacidin induced the upregulation response of *Tp* cells on the silacidin gene, which caused the overexpression of silacidin when the protease activities of *Alteromonas* decreased with time and therefore shrank the cell size. To our knowledge, this potential survival mechanism of diatom by regulating the cell size has never been reported before. It might be a cell-specific strategy responding to the swarming behavior of lytic bacteria.

**Fig 7:**
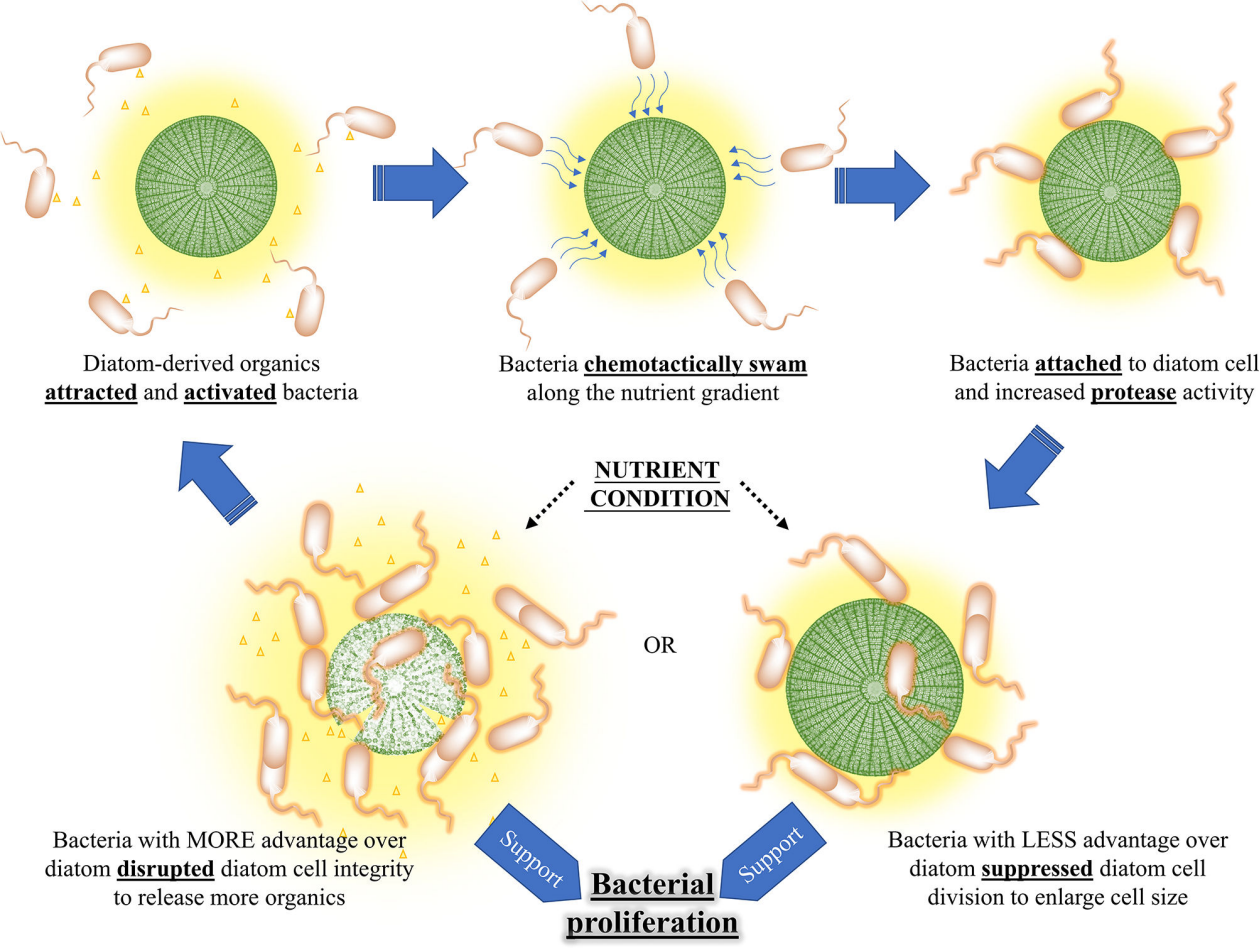
Contact-dependent algicidal mechanism of L15 against *Tp* as a chain reaction.

More importantly, the contact-dependent algicidal mechanism controlled by the nutrient condition is ecologically significant. In fact, the involvement of algicidal bacteria in the demise of natural algal blooms is elusive (44). The paradox is, for those metabolite-dependent algicidal bacteria, producing enough algicides in the diffusive marine environment would be metabolically costly (36), which may occur only at the decay phase of algal blooms when nutrients are sufficient (15). Combining with the fact that bacteria growth in bloom areas usually starts right after algal decline (1) and the secretion of algicides is often controlled by quorum sensing (15), these known algicidal bacteria would have difficulty in inducing the demise of algal blooms and could only accelerate the final stage. In contrast, the algicidal process we proposed was a chain reaction that coupled the changes of nutrient viability, bacterial proliferation, and algal demise: a few bacteria that sensed and became activated by limited diatom-derived organics chemotactically swam along the nutrient gradient; bacteria then attached to the surface of diatom cells, where bacterial protease activity was induced; proteases caused the enlargement or leakage of diatom cells, which provided more nutrients for bacterial proliferation; the increase of attached bacteria strengthened the algicidal intensity and promoted the release of diatom-derived organics, resulting in attracting more algicidal bacteria (Fig. 7). This hypothetical algicidal process with spatial-temporal dynamics linearly linked to the nutritional level fits the real world better and might actually trigger the collapse of algal blooms. It highlights the ecological importance of these chemotactic copiotrophs and suggests their great contribution to the massive release of alga-derived organics, which is opposite to the traditional opinion treating the dominance of copiotrophs as the outcome of organic enrichment after bloom collapsed (45).

Extrapolating from our laboratory studies, we envision that algicidal copiotrophs could greatly impact marine carbon cycles since they would be entrained into algal aggregates as marine snow. In such “infected” marine snow, algicidal copiotrophs thrive, disperse, and cause extensive breakdown of marine snow if detached bacteria infected surrounding aggregates, leading to DOM release too rapid for bacterial uptake (46). Such conversion of sinking POM to non-sinking DOM would reduce the efficiency of the biological carbon pump (BCP). These hypothetical scenarios are also relevant to a discussion of ocean fertilization (e.g., iron enrichment) to generating algal blooms for stimulating fish production and the activity of the BCP (47), which may result in increasing the contribution of the microbial carbon pump (MCP) to carbon sequestration instead (48). Generally, raise of the MCP:BCP ratio is correlated with increased microbial respiration and decreased phytoplankton mean size (49, 50), which implicates higher activity of microbial transformation of DOC and lower exportation of primary production through gravitational sinking, respectively. These phenomena were usually ascribed to the oligotrophic surface seawater caused by nutrient stratification, which would increase the MCP:BCP ratio up to six times (1.5 times if predation was not considered) (51). Differently, our study suggests that the higher MCP:BCP ratio caused by increased bacterial activity and decreased phytoplankton size can be also a result of intensive bacteria-phytoplankton competitions occurring in bloom areas, which may overturn current predictions on carbon sequestration pathways in the eutrophic environment if the hypothesis was fully verified *in situ*. In summary, the ecological role of algicidal copiotrophs should be re-examined for their influence on phytoplankton community succession and marine carbon cycles.

## SUPPLEMENTAL MATERIAL

Supplemental methods and Fig. S1 to S12 mbio.00940-23-s0001.docx Additional experimental details and supplemental figures.

Table S1 and S2 mbio.00940-23-s0002.xlsx Protease composition of *Alteromonas macleodii*.

Video S1 mbio.00940-23-s0003.mp4 Time-lapse video showing the morphological change of Tp cells under the attack of L15 within 17 min.

Video S2 mbio.00940-23-s0004.mp4 Real-time video recorded 1 min and 10 min after the addition of L15.

Video S3 mbio.00940-23-s0005.mp4 Time-lapse video showing the integrity of Tp cells without (Left)/with (Right) the influence of L15 within 60 min.

ASM does not own the copyrights to Supplemental Material that may be linked to, or accessed through, an article. The authors have granted ASM a non-exclusive, world-wide license to publish the Supplemental Material files. Please contact the corresponding author directly for reuse.
