# Supplementary material for: Nutrient-dependent interactions between a marine copiotroph Alteromonas and a diatom Thalassiosira pseudonana
Source: mBio. 2023 Sep 29;14(5):e00940-23. doi: 10.1128/mbio.00940-23 (PMC10653928; doi:10.1128/mbio.00940-23)
Supplement: Supplemental methods and Fig. S1 to S12 — Additional experimental details and supplemental figures. [file mbio.00940-23-s0001.docx]

Nutrient-dependent interactions between a marine copiotroph *Alteromonas* and a diatom *Thalassiosira pseudonana*

Guanjing Cai^a,b^^[[1]](#footnote-1)^*, Xiaoqi Yu^b^, Hui Wang^a^, Tianling Zheng^b^, Farooq Azam^c^

*^a^ Biology Department and Institute of Marine Sciences, College of Science, and Guangdong Provincial Key Laboratory of Marine Biotechnology, Shantou University, Shantou 515063, China*

*^b^ State Key Laboratory of Marine Environmental Science and Key Laboratory of the Ministry of Education for Coastal and Wetland Ecosystems, School of Life Sciences, Xiamen University, Xiamen 361005, China*

*^c^ Marine Biology Research Division, Scripps Institution of Oceanography, University of California, San Diego, La Jolla, CA 92093, USA*

***Contents:***

***Supplementary Methods***

***Supplementary Figures (S1-S12)***

***Supplementary Videos (S1-S3)***

# Supplementary Methods

1. **Counting of bacterial and diatom cells, fitting of growth curve and calculation of algicidal rate**

Samples of FASW-based groups were stained with LIVE/DEAD Baclight Bacterial Viability kit and analyzed with the flow cytometer based on FITC/PerCP and APC/FSC signals to distinguish and count the live and dead cells. In detail, live cells only had signals in FITC channel (Ex/Em=480/500 nm) while dead cells showed strong signals in PerCP channel (Ex/Em=490/635 nm). Diatom cells were further distinguished from bacterial cells based on the APC signals (Ex/Em=650/660 nm, Chlorophyll fluorescence) and higher FSC signals (larger cell size). Samples of F/2-based groups were stained with SYBR Green I and counted for cells with the flow cytometer. Each sample was measured at least twice to reduce random errors. The growth curves of L15 and *Tp* cells in each experimental group were fitted by the Gompertz model(1):

$$Ln\left( \frac{N_{t}}{N_{0}} \right)=A\cdot e^{-e^{\left[ \left( U_{max}\cdot\frac{e}{A} \right)\left( \lambda-t \right)+1 \right]}}$$

where N_t_ and N_0_ are the cell counts at time t and initial, respectively; A is the maximum cell count during the stationary phase; U_max_ is the maximum specific growth rate; $\lambda$ is the lag time. Data of late stationary phase of L15 (after 48 h) were discarded to increase the fitting accuracy.

Algicidal rate (A) was calculated using the equation below.

$A\left( \% \right)=\left( 1-\frac{C}{M} \right)\times100\%$,

Where C and M stand for the live diatom counts in the co-culture and the monoculture of *Tp* under the same nutrient condition, respectively. All experiment groups were triplicated.

1. **Counting of attached bacteria and measurement of diatom cell lengths**

Samples of “Co-culture”, “Co+Z/1000” at 48, 72, 78, 144 h and “Co*+Z/100” at 24, 48, 72, 144 h were stained with 1 μM 4',6-Diamidino-2-Phenylindole (DAPI) for 10 min before cells were collected by gentle filtration on 0.22 μm filters. The attached bacteria per diatom cell were counted under an epifluorescence microscope (Nikon D-Eclipse C1 Confocal Microscope) based on DAPI and TRITC (to identify diatom cells) signals. The lengths of diatom cells in “Co*+Z/100” with at least one attached bacterial cell were also measured by double inspection based on bright field and fluorescence, to see the correlation between the number of attached bacteria and cell size.

1. **Measurement of nutrient concentrations**

At 0, 24, 72 and 144 h, samples of FASW-based groups were collected and filtered through GF/F. The filtrates were measured for NO_3_^-^, NO_2_^-^, NH_4_^+^, PO_4_^3-^ and dissolved organic carbon (DOC) content using Seal Analytical^®^ Continuous-flow AutoAnalyzer 3 and Shimadzu^®^ 500 V-CSN/TNM-1 TOC Analysis System.

1. **Measurement of swimming velocity and chemotactic movement**

The movement of bacteria was observed with a Nikon Eclipse Ti-E inverted microscope equipped with a CCD camera (Andor Clare) under a bright field. The field of view was 10 mm away from the inlet with the midline overlapped with the boundary between bacterial and attractant fluid, as shown in Figure S3(B). To measure the velocity of bacteria, the videos were taken at 11.4 fps for 10 s after the fluids completely stopped for 5 min. To see whether bacteria swam toward the attractants, pictures were taken immediately after the fluids stopped (31 frames in 10 min) and the whole procedures were replicated for four times. All videos and pictures were preliminarily processed in NIS-elements AR 4.10. Bacteria in the images were further identified, positioned, counted and tracked using a script based on the Image Processing Toolbox running on MATLAB R2014b. The velocity of each bacterial cell at each time interval (87.7 ms) was calculated by dividing displacement distance by time. The chemotactic enrichment of bacteria was characterized by the percentage of bacteria in attractant, which was calculated by dividing bacterial count in attractant fluid by whole bacterial count in the field of view.

The chemotactic movement of bacteria towards a single *Tp* cell was measured one minute after the fluids completely stopped. 90 frames were then taken in 30 min. To avoid the disturbance of non-motile particles or bacterial cells, only bacteria with displacement were identified, positioned and counted, then categorized based on their distance away from the *Tp* cell. Bacterial densities in different ranges (<50 μm or 50~150 μm) were calculated by dividing the cell counts by the area of the corresponding circles (7.85×10^3^ and 6.28×10^4^ μm^2^, respectively).

1. **Calculation of cell-specific protease activity**

Substrate hydrolysis rates were measured with SpectraMax M2 microplate reader (excitation 355 nm, emission 460 nm) and calculated with the standard curve of 7-amido-4-methylcoumarin. Cell-specific protease activity of L15 was calculated by dividing the activity of bulk filtrate (for attached bacteria, it equals the activity of unfiltered culture minus 5 μm filtrate; for free-living bacteria, it equals the activity of 5 μm filtrate minus 0.22 μm filtrate) by corresponding bacterial cell density (the activity of axenic *Tp* was negligible). Monoculture of *Tp*, as well as the aliquots amended with 1% Zobell medium and PMSF, were used as control groups to calculate the algicidal rates. All experiment groups were triplicated.

**Reference**

1. Zwietering MH, Jongenburger I, Rombouts FM, van 't Riet K. 1990. Modeling of the Bacterial Growth Curve. Appl Environ Microbiol 56:1875-1881.

# Figure S1. Phylogenetic tree based on the 16S rRNA gene sequence.


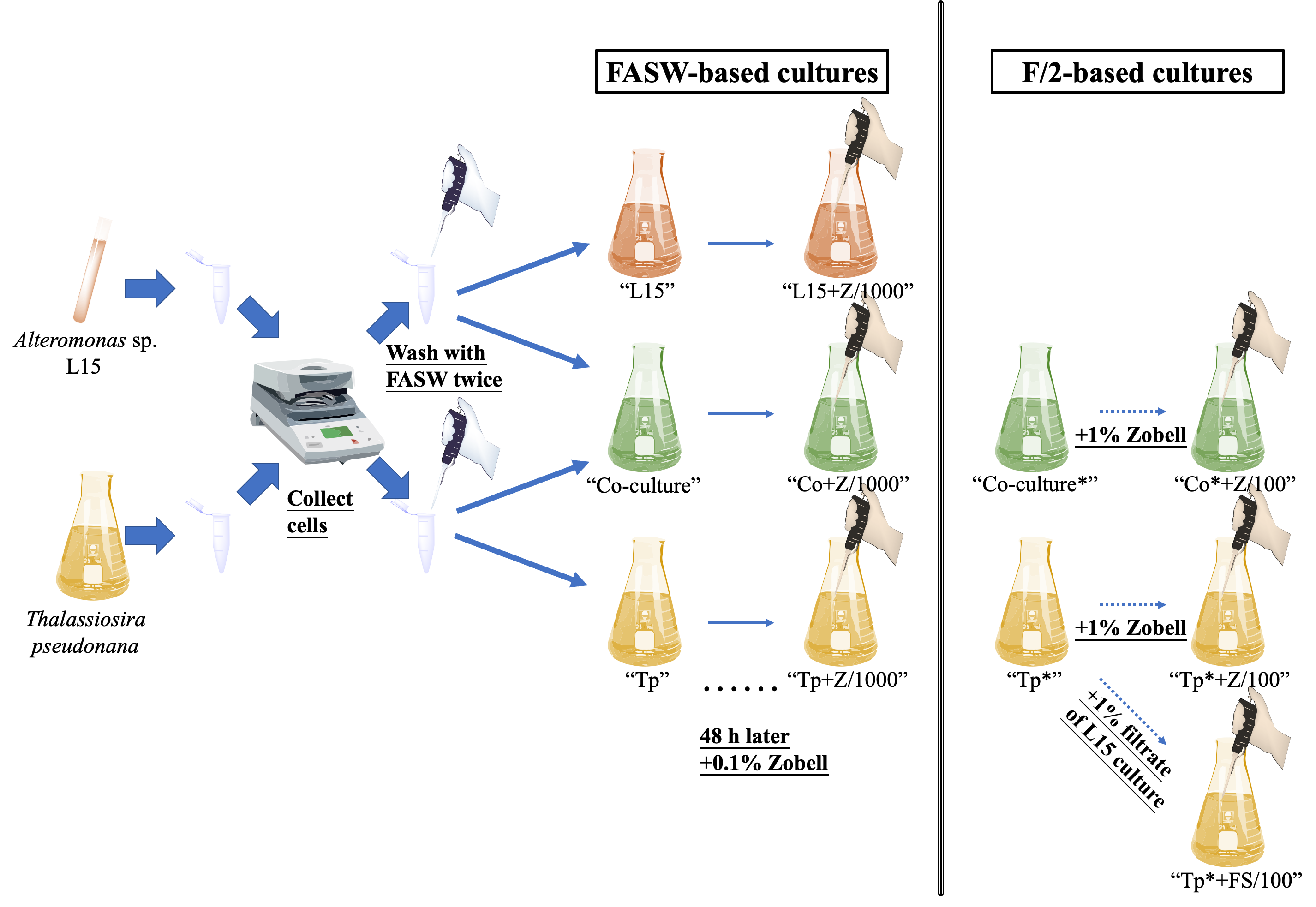


# Figure S2. Experimental setups for long-term co-cultivation.

# Figure S3. Schematics of the PDMS microchannel (A) and the relative position of fluids seen under the microscope (B).

# Figure S4. Schematic of counting the free-swimming bacteria with different distance to the diatom cell. Radius of red circle: 50 μm; green circle: 150 μm.


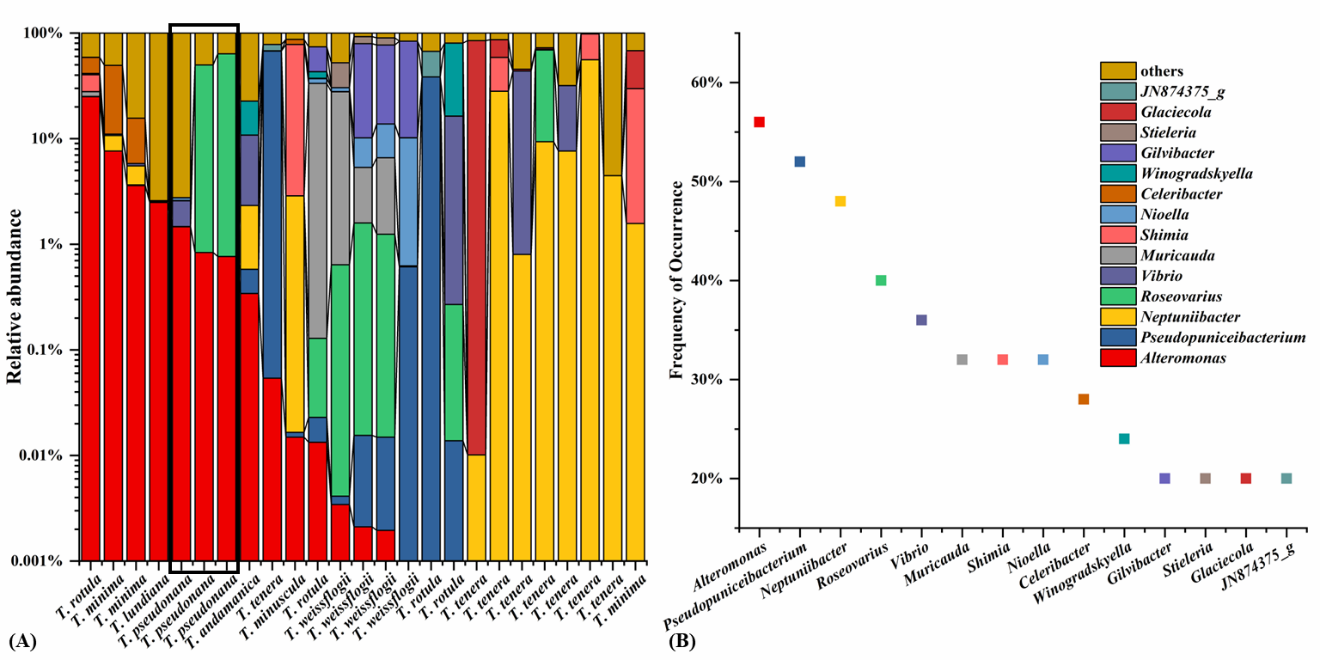


# Figure S5. Bacterial community compositions of 25 *Thalassiosira* samples *in situ* (A) and frequencies of occurrence of top 14 bacterial genera (B). The black box marks the *T. pseudonana* samples. All samples were collected from coastal waters of China (unpublished data). Single diatom cells were picked out under microscope and identified based on the 18S rRNA gene. The V4-V5 regions of the 16S rRNA gene from the diatom samples were sequenced via pair-end Illumina MiSeq platform to reveal the compositions of bacterial communities in the phycosphere.


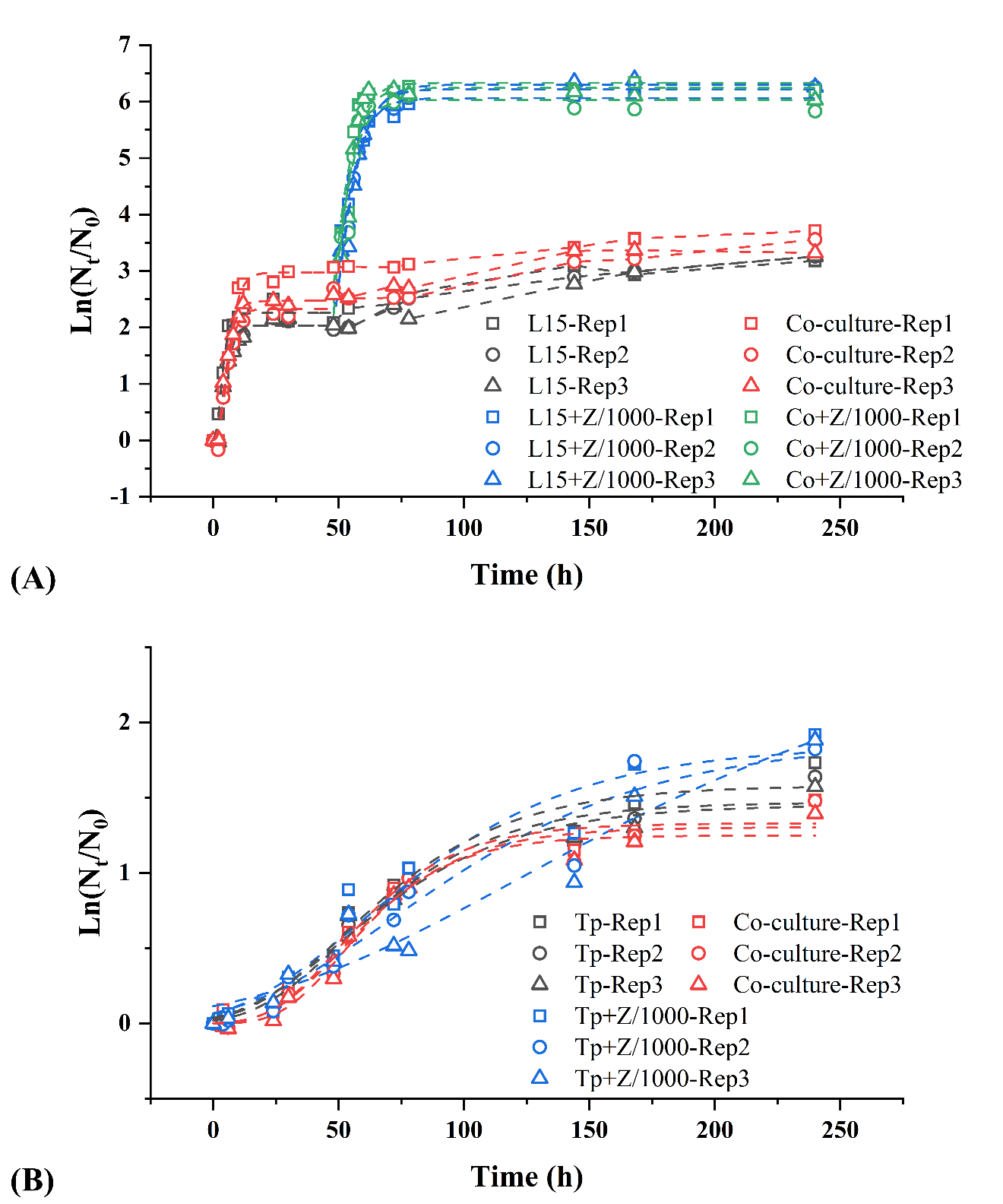


# Figure S6. Growth curves of L15 (A) and *Tp* (B) in FASW fitted by Gompertz model.

**
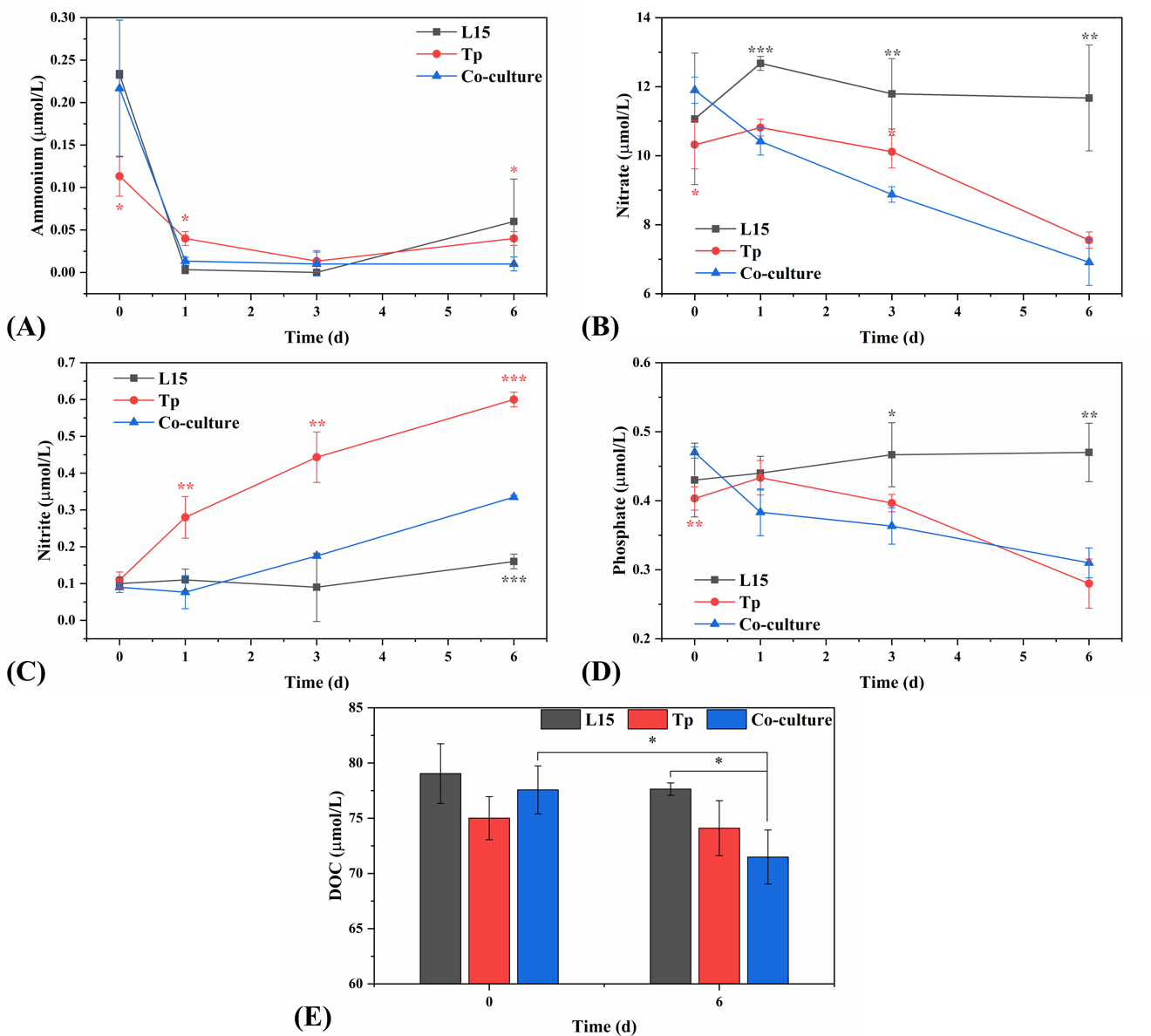
**

# Figure S7. Time-variation of ammonium (A), nitrate (B), nitrite (C), phosphate (D) and DOC (E) in the co-culture of L15 and *Tp*. The black or red asterisk represents that the co-culture is statistically different from the monoculture of L15 or *Tp* (t-test, *: 0.01<p<0.05, **: 0.001<p<0.01, ***: p<0.001).





# Figure S8. Growth curves of L15 under different nutrient conditions.





# Figure S9. Co-culture of L15 and *Tp* in F/2 medium with different initial bacterium/diatom ratios. The dots and columns in the chart above represented the bacteria and diatom counts in the co-culture, respectively. The dots and columns in the chart below represented the bacteria/diatom ration and inhibitory rate on diatom growth, respectively.

**1×L15+1×Tp**: initial L15 density: 5×10^6^ mL^-1^, initial *Tp* density: 3×10^4^ mL^-1^, no Zobell; **4×L15+1×Tp**: initial L15 density: 2×10^7^ mL^-1^, initial *Tp* density: 3×10^4^ mL^-1^, no Zobell; **1×L15+1×Tp+Z/100**: initial L15 density: 5×10^6^ mL^-1^, initial *Tp* density: 3×10^4^ mL^-1^, 1% Zobell, a bacterium/diatom ratio close to **4×L15+1×Tp** at 3 h; **1×L15+3×Tp+Z/100**: initial L15 density: 5×10^6^ mL^-1^, initial *Tp* density: 10^5^ mL^-1^, 1% Zobell, a bacterium/diatom ratio close to **1×L15+1×Tp** at 3 h.


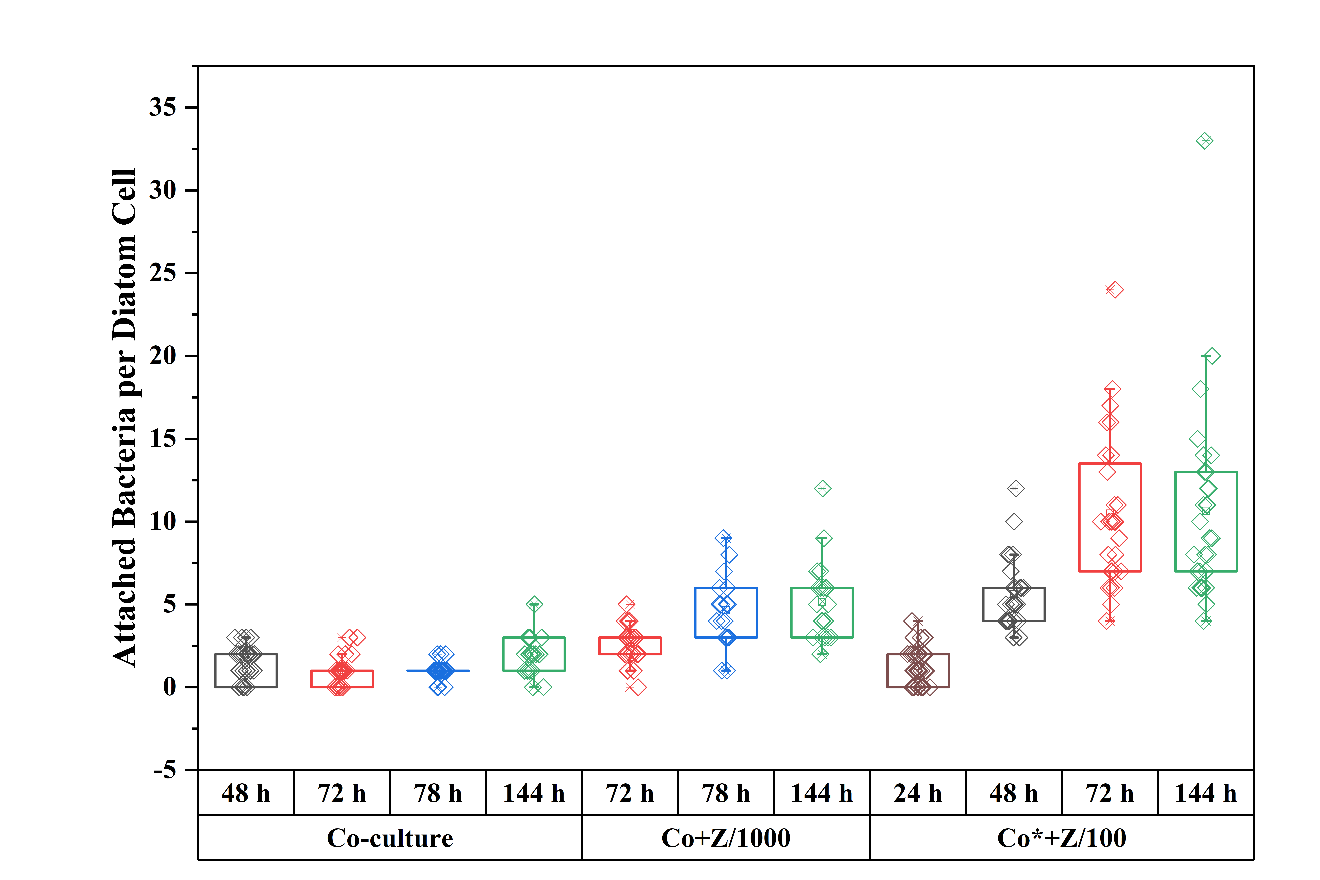


# Figure S10. Summary of attached L15 cells on the surface of *Tp* cells.


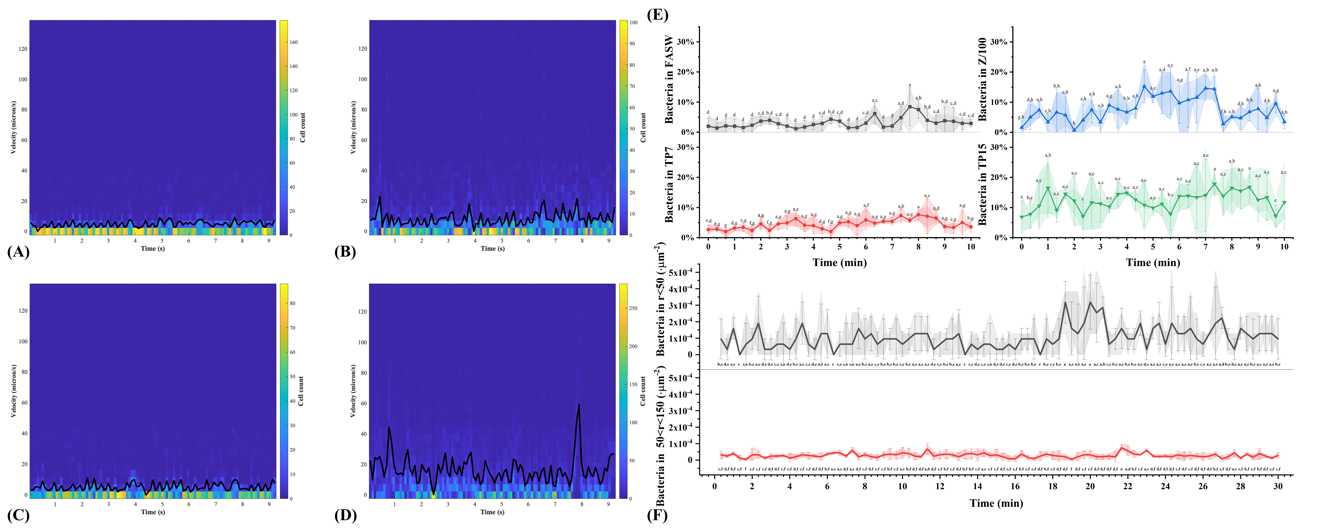


# Figure S11. Swimming velocities of L15 stimulated by FASW (A), 1% Zobell medium (B), cell-free medium of *Tp* culture grown for 7 d (C) and 15 d (D), respectively. The thick line inside each diagram showed the variation of average velocity at each time interval. The percentages of bacteria in different fluids and the bacterial densities in different ranges away from *Tp* cells were shown in E and F. Shadow areas were enclosed by the curves of the four replicates. Data points marked with different letters were significant different from each other (p<0.05, one-way ANOVA, Duncan’s multiple range test)


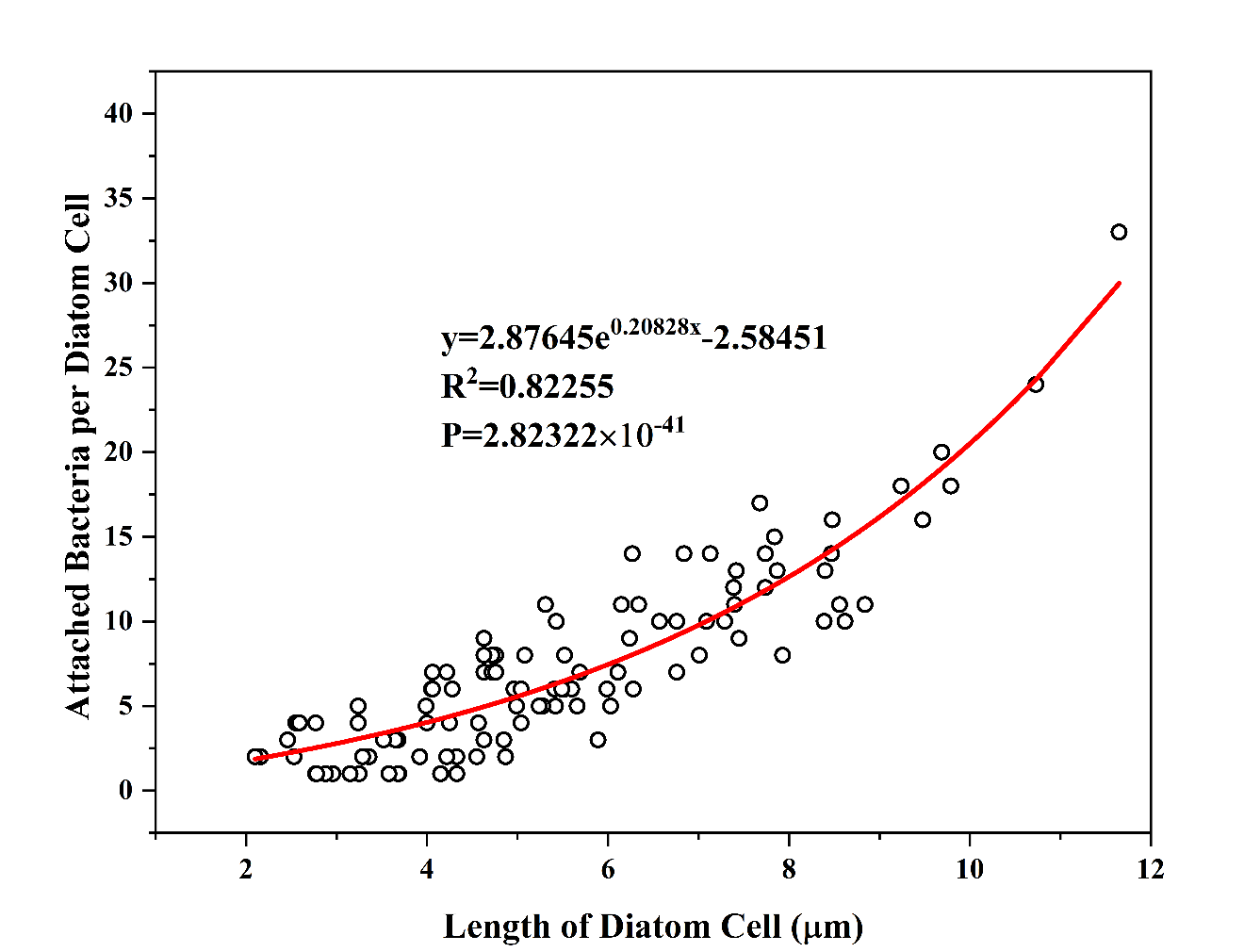


# Figure S12. The exponential fit between attached bacteria per diatom cell and length of diatom cell.

# Video S1. Time-lapse video showing the morphological change of *Tp* cells under the attack of L15 within 17 min.

# Video S2. Real-time video recorded 1 min and 10 min after the addition of L15.

# Video S3. Time-lapse video showing the integrity of *Tp* cells without (Left)/with (Right) the influence of L15 within 60 min.

1. * *Corresponding author*

   Email: [gjcai@stu.edu.cn](mailto:gjcai@stu.edu.cn) Tel: +86-15659294569 [↑](#footnote-ref-1)
